# Optimizing the Delivery of Inhaled Medication for Respiratory Patients: The Role of Valved Holding Chambers

**DOI:** 10.1155/2018/5076259

**Published:** 2018-04-04

**Authors:** R. Andrew McIvor, Hollie M. Devlin, Alan Kaplan

**Affiliations:** ^1^St. Joseph's Healthcare Hamilton, McMaster University, Hamilton, ON, Canada; ^2^Hollie Devlin Consulting, Toronto, ON, Canada; ^3^Family Physician Airways Group of Canada, Richmond Hill, ON, Canada; ^4^Department of Family and Community Medicine, University of Toronto, Toronto, ON, Canada; ^5^Health Quality Ontario COPD Community Standards Project, Toronto, ON, Canada

## Abstract

Valved holding chambers (VHCs) have been used with pressurized metered-dose inhalers since the early 1980s. They have been shown to increase fine particle delivery to the lungs, decrease oropharyngeal deposition, and reduce side effects such as throat irritation, dysphonia, and oral candidiasis that are common with use of pressurized metered-dose inhalers (pMDIs) alone. VHCs act as aerosol reservoirs, allowing the user to actuate the pMDI device and then inhale the medication in a two-step process that helps users overcome challenges in coordinating pMDI actuation with inhalation. The design of VHC devices can have an impact on performance. Features such as antistatic properties, effective face-to-facemask seal feedback whistles indicating correct inhalation speed, and inhalation indicators all help improve function and performance, and have been demonstrated to improve asthma control, reduce the rate of exacerbations, and improve quality of life. Not all VHCs are the same, and they are not interchangeable. Each pairing of a pMDI device plus VHC should be considered as a unique delivery system.

## 1. Introduction

Inhaled therapy is the gold standard for treatment of asthma [1] and chronic obstructive pulmonary disease (COPD) [2] in patients treated with pressurized metered-dose inhalers (pMDIs). The first pMDI was introduced in 1956 by Riker Laboratories Inc. and constituted a significant advancement in the delivery of aerosol medication [3]. pMDIs are now the most widely used devices for the delivery of aerosol medication because of their low cost, effectiveness, and relative simplicity of use [4]. However, despite instruction, many patients are unable to use their pMDIs correctly, with the most common error being the inability to synchronize actuation with inhalation [5].

**Figure 1:**
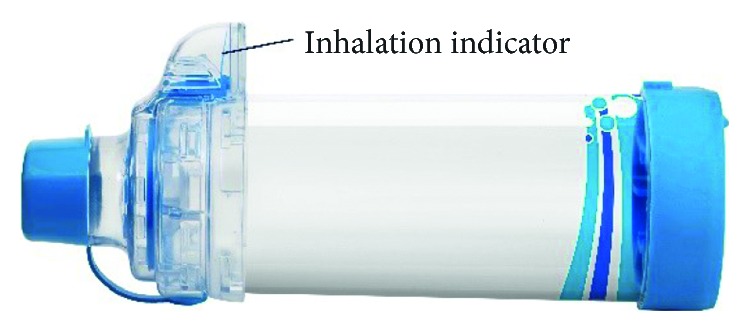
Valved holding chamber featuring an inhalation indicator.

Spacer devices were introduced in 1958 to improve medication delivery into the lungs and reduce deposition in the mouth and throat [3]. Spacers have undergone a significant transformation since their origin as simple home-made tubes such as toilet paper tubes, plastic cups, and empty vinegar bottles [3]. In 1981, both cone and tube spacers were proven to improve medication deposition on the conducting airways without changing alveolar deposition [6]. Around the same time, the valved holding chamber (VHC) was introduced. The VHC built on the spacer design by adding a one-way inhalation valve at the exit of the chamber which traps the aerosolized medication within the chamber until the user inhales. VHCs improve pMDI medication delivery, reduce oropharyngeal deposition of medication, and help users overcome challenges in coordinating pMDI actuation with inhalation [7–9]. In 1994, Holzer and Muller [10] showed that valve construction had an impact on the delivery of medication. Subsequent to 1994, VHCs were further improved with respect to facemask design in order to obtain a better face-to-facemask seal, incorporation of antistatic materials to reduce attraction of medication to the chamber walls, and feedback features such as whistles which indicate correct inhalation speed and inhalation indicators (Figure 1).

In 2009, the European Medicines Agency recognized the significant role of the VHC in drug delivery when they recommended that the development of drug products using a pMDI include testing with at least one named VHC. In addition, they recommended that if a VHC was to be substituted or added, appropriate equivalency data for the alternative VHC must be presented [11].

Current guidelines for asthma [1, 9] and chronic obstructive pulmonary disease (COPD) [2] mention the use of a spacer in conjunction with pMDIs but do not discuss VHCs in great detail or provide much guidance on their use for health-care providers or caregivers. The American Academy of Allergy Asthma and Immunology (AAAAI) states that VHCs with whistles and one-way valves are preferable to spacers [12].

## 2. pMDIs and the Challenges of Inhaler Technique

Inhaled therapies can be very effective treatment for lung diseases. However, they must be used correctly for patients to obtain their full benefit. Poor inhaler technique is common with all types of inhalers and has been examined extensively [5, 13, 14]. A review of 2123 asthma patients by the National Services for Health Improvement found that without training, 86% failed to properly use their inhaler [15]. Another study showed that only 1 out of 10 patients using an MDI were able to perform all essential steps correctly [16]. Even with inhaler training, some people will revert back to bad technique, and some will not benefit from training [17]. The most common errors associated with pMDIs are the lack of coordination between actuation and inhalation; halting inhalation when the cool spray hits the back of the throat; not holding the breath long enough (>5 seconds) after inhalation; no exhalation prior to actuation; and not shaking the suspension prior to use [13, 18]. A recent literature review indicates that inhaler technique has not improved over the past 40 years [19].

Implications of poor technique include unintentional noncompliance (patient is compliant but has poor inhaler technique) [14], significantly reduced efficacy of the medications [5, 13, 18], increased risk of hospitalizations and emergency room visits, and poor disease control [13]. Giraud and Roche [18] showed that 71% of adult asthmatic patients are unable to use their inhaler devices effectively, resulting in decreased control of asthma symptoms. All of these lead to increased health-care expenditure [20]. Factors that impact the ability of patients to correctly use inhalers include age, coordination, inhaler technique training, choice of inhaler type, and use of multiple inhalers [2].

Additional challenges arise when patients are prescribed multiple inhaler devices that require different inhalation techniques. While pMDIs require a slow and deep breath, dry powder inhalers (PDIs), and breath-actuated metered-dose inhalers (BA-MDI) require a fast and deep inhalation [20, 21]. Multiple inhaler users compared to single inhaler users have been shown to experience significantly more exacerbations, have a higher risk of exacerbations, higher rates of inpatient admissions, inpatient days, urgent care visits, and outpatient visits [22].

Patients using two or more inhaler types that require different inhaler techniques are prone to a higher frequency of errors and have impaired outcomes. Bosnic–Anticevich et al. [23] showed lower rates of exacerbation and reduced use of high doses of short-acting beta agonists in COPD patients requiring more than one inhaler when the inhalers require similar inhalation techniques compared to inhalers that require different inhaler techniques.

Challenges resulting from poor inhaler technique often start at the point of prescription. The clinicians responsible for teaching inhaler technique, including nurses, doctors, and respiratory therapists, are often unable to describe or perform the critical steps for using inhalers [24, 25]. One study showed that only 14.2% of >1500 physicians who frequently prescribe medications delivered by inhaled devices had adequate knowledge of inhaled therapy, including correct use of inhalers [26].

## 3. Valved Holding Chambers (VHCs)

**Figure 2:**
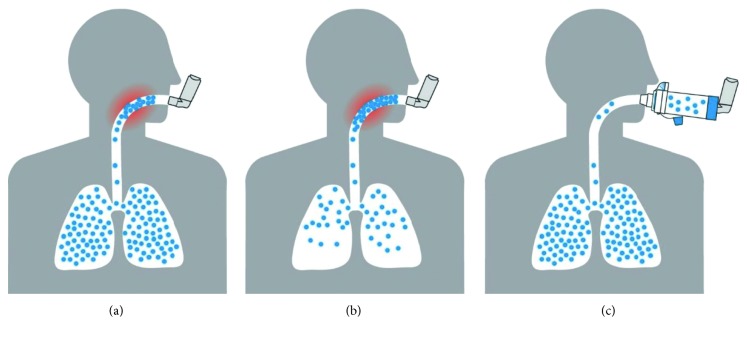
Lung deposition of inhaled medication is improved with perfect pMDI technique and with valved holding chamber. (a) Inhaler alone with perfect technique. (b) Inhaler alone with poor technique. (c) Inhaler with valved holding chamber.

A key challenge with pMDIs is the potential for unwanted deposition of drug and excipients in the mouth and the back of the throat. This remains an issue with hydrofluoroalkane (HFA) formulations and to some extent with slower, finer spray products [27, 28]. Spacer devices and VHCs were initially designed to increase delivery of aerosol medications to the lungs while reducing the oropharyngeal deposition [3]. The space between the pMDI and the user's mouth allows for a reduction of aerosol velocity and particle size through both evaporation and particle impaction on the spacer wall. Together, this leads to an increase in the proportion of fine particles delivered to the lungs, a decrease in large particle deposition in the mouth and throat [29] (Figure 2) and reduction of associated side effects such as throat irritation, dysphonia, and oral candidiasis that are common with use of pMDIs alone [4, 29]. VHCs act as aerosol reservoirs, allowing the user to actuate the pMDI device and then inhale the medication in a two-step process that reduces the need to coordinate actuation and inhalation at the same time [30]. This is a particularly important advantage of using VHCs with pMDI devices.

The design of the device can have an impact on its performance. Throughout the development of VHCs, it was recognized that while large-volume holding chambers increase lung deposition to a greater degree than tube spacers or small holding chambers, they could be cumbersome for patients. Currently available devices range in volume from 50–750 ml [4]. Larger VHCs may be less portable and appealing, especially among children and teens, which may impair adherence [4]. Chambers with volumes from 150 to 250 ml have been shown to be as effective as those with volumes of 750 ml [31]. Smaller chamber sizes are generally more effective for infants and small children as they require fewer tidal breaths to empty. One brand introduced in 1983 was the result of a design study to determine the optimal size of chamber, focusing on minimizing the size of the chamber without significantly reducing fine particle output [8].

Roller et al. [32] found that specifically for children (to ages 17 years) using VHCs with pMDI devices, slow inhalation and breath hold (5–10 seconds) may be more effective than tidal breaths for lung deposition of extra-fine aerosols for users who are capable of this type of breathing. For younger children or individuals with less ability to control their breathing (e.g., delayed or impaired development), tidal breathing is appropriate. Oropharyngeal and gastrointestinal deposition is reduced using VHCs regardless of inhalation technique.

VHCs fitted with facemasks are useful for infants, young children [1], and elderly patients [4, 31, 33]. There are various facemask designs available. The most important features for optimum aerosol delivery are a tight but comfortable facemask fit and reduced facemask dead space [30]. Esposito-Festen et al. [34] demonstrated that the efficiency of a pMDI-spacer facemask strongly depends on the size and location of facemask leaks. Minor leaks can reduce the lung dose by half compared to lung dose received with a facemask with a perfect seal, and leaks near the nose result in more rapidly decreased lung dose compared to leaks near the chin. A flexible facemask that conforms to the user's face to prevent leakage is optimal [31].

The valves of VHCs ideally allow the aerosolized medication to remain in the chamber until inhalation and the patient to exhale to the atmosphere without blowing the aerosol out of the chamber. Valve design and placement impact the rebreathed volume of the device, which is especially critical for use with infants and young children [20]. Valves are usually the only moving part of a VHC and are made from flexible components. Users should be taught to inspect the valve integrity when cleaning their VHC devices.

Some plastic VHC devices have an inherent electrostatic charge or may build up electrostatic charge within the chamber, which attracts drug particles to the chamber walls and may reduce drug delivery to the lungs [29]. Electrostatic charges may also result in inconsistent delivery of the medication [30]. Drug delivery through the use of antistatic chambers can provide clinically relevant improvement in bronchodilator response during acute, reversible bronchospasm such as nocturnal bronchospasm [35]. Researchers found that forced expiratory volume in 1 second (FEV1) increased by 21–25% when using an antistatic chamber and concluded that delivery of albuterol through an antistatic chamber provides a clinically relevant improvement in bronchodilator response during acute, reversible bronchospasm [35]. More recently, Burudpakdee et al. [36] examined a retrospective study database and identified 9325 patients with asthma in each of two groups: those using a pMDI with the AeroChamber Plus® Flow-Vu® Antistatic Valved Holding Chamber (AC-FV AVHC) and those using pMDI with any nonantistatic VHC (control group). The use of the AC-FV AVHC was associated with lower exacerbation rates, delayed time to first exacerbation, and lower exacerbation-related costs when compared to control nonantistatic VHCs. Further, the proportion of patients visiting the emergency department (ED) was significantly lower in the AC-FV AVHC group than the control cohort (10.8% versus 12.4%; *p* < 0.05), and the number of ED visits per patient was also significantly lower (0.15 visits versus 0.18 visits for the control VHC; *p* < 0.05).

Electrostatic charge within a VHC can also be reduced somewhat by priming the device with multiple actuations of the aerosol medication, but the number of actuations depends on the content of the aerosol and can be wasteful of medication. Alternately, washing the chamber with a mild detergent without rinsing and then allowing it to drip dry has proven more effective. The charge may reaccumulate after about 30 days [20, 31, 37].

Both for hygiene reasons and to prevent deterioration in valve function, spacers should be washed on a regular basis [38]. Each spacer has its own particular recommended washing and use procedures, and so it is important to follow the instructions in the Patient Information Leaflet (PIL). Spacers made of antistatic materials or metals such as steel or aluminum are less subject to this problem and therefore, with the incorporation of an effective antistatic material, remove the need for washing with detergent prior to first use [31].

## 4. Use of pMDIs with and without VHCs

Many studies have assessed disease control and oropharyngeal deposition using pMDIs with and without spacers or VHCs. Leach and Colice [28] conducted a small pilot study which showed that both the AeroChamber™ and Volumatic™ devices used in the study reduced oropharyngeal deposition of HFA-134a-beclomethasone dipropionate (BDP) and CFC-BDP. They found that oropharyngeal deposition of HFA-BDP was reduced from approximately 28% to 4% with the AeroChamber. Roller et al. [32] demonstrated a higher lung deposition and marked reduction in oropharyngeal deposition of BDP extra-fine particles when delivered via an HFA-driven inhaler with an attached VHC compared with delivery via the HFA-driven inhaler alone. Levy et al. [5] found better asthma control in significantly more patients using BDP through a breath-actuated pMDI device (*p* < 0.0001) or a spacer (*p* < 0.0001) compared with those using pMDIs alone.

Several studies have assessed the efficacy and cost-effectiveness of treating moderate and acute asthma exacerbations in children and adults in the ED setting. The use of pMDI plus spacer delivers equal or better clinical outcomes than nebulizers, with significant savings in costs and health-care provider time compared to nebulizers [39–43].

The majority of studies assessing the effectiveness of using VHCs with pMDIs are conducted under clinical trial settings, with careful training of participants in the devices used, and monitoring to ensure adherence of both medication use and correct use of devices. Guilbert et al. [44] reviewed anonymous medical record data from UK databases of patients with asthma prescribed fine and extra-fine particle ICS with and without spacers. They found no difference in rate of severe exacerbations and acute respiratory events. The authors recognize that the real-life nature of this study data means that the patients were not chosen through highly selective processes that are normally included in clinical trial designs. They were unable to determine if patients had been taught to use their devices correctly, whether or not they used them correctly, or in fact if the patients were adherent to treatment (i.e., did they use medications as prescribed, and did they use the spacers).

## 5. VHCs Are Not Interchangeable

VHCs are available in various sizes (Table 1) and, as has been described earlier, incorporate attributes such as valving, chamber shape/material, and facemask size and seal, each can have an impact upon drug delivery. Each pairing of a pMDI device plus VHC should be considered as a unique delivery system. Suggett et al. [45] assessed six VHCs and their associated facemasks for their ability to deliver fine particles in an *in vitro* simulated child model. They demonstrated significantly more medication delivery from the AeroChamber Plus^∗^ Flow-Vu^∗^ VHC compared with any of the other antistatic devices evaluated, and regardless of device pretreatment modality. Blake et al. [46] examined the lung bioavailability of fluticasone propionate pMDI administered through AeroChamber Plus with facemask and Babyhaler® VHCs in children from 1 to 4 years of age. Clinically significant differences in lung bioavailability were observed between the devices. Based on the study results, the authors recommend that parents, clinicians, and pharmacists should be educated not to interchange VHCs once a child is stable on a particular ICS dose and VHC combination and that VHC prescriptions should include a no substitutions statement. A recent study [47] reported an *in vitro* statistical equivalence study comparing a number of different, similarly sized VHCs in terms of particle size distribution of the medication delivered. The study demonstrated that only the two AeroChamber Plus VHC variants were equivalent; all the other VHCs tested were both statistically and likely clinically different (i.e., lower fine particle mass).

Suggett et al. [48] used an *in vitro* model to assess the difference between two antistatic and three nonconducting VHCs that look similar, using the AeroChamber Plus^∗^ Flow-Vu^∗^ as the reference. Devices were tested before and after following manufacturer-recommended washing procedures. The influence of wash on the fine particle mass delivered from the antistatic devices AeroChamber Plus^∗^ Flow-Vu^∗^ and Pocket Chamber was minimal but was significant on the three nonconducting VHCs for which a prewash was required to mitigate the effect of static charge and resultant reduced fine particle mass. Fine particle mass and total emitted mass (under simulated coordinated and uncoordinated use) were examined and statistically compared. All devices showed significantly different (reduced) results compared to reference.

## 6. VHC Approval Process

The European Medicines Agency (EMA) recommends that the development and registration process for new pMDIs includes testing and supporting data (*in vitro* and *in vivo*) of the pMDIs when used with specific VHC devices [49]. A separate communication stated that patients whose asthma is well controlled and who are using a spacer should always use the same type of spacer and not switch between spacers [50]. The EU registration documentation for pMDIs (Summary of Product Characteristics and Patient Information Leaflet) specifically note the VHC to be used should be based upon the device used in the product development/registration studies. For example, in Europe, the following pMDI products recommend the AeroChamber Plus brand of VHCs: Airomir, AirSalb, Alvesco, Atrovent, Flutiform, Fostair, Qvar, Seretide, and Sirdupla. The EMA is alone in requiring data for a “*specific named spacer*” in order to support pMDI approval generally. The US Food and Drug Administration (FDA), Health Canada, and Japan's Pharmaceuticals and Medical Devices Agency are silent on this issue. As was stated by Dissanayake and Suggett [47], the FDA's position is perhaps the most surprising in view of the agency's zealous approach to dose selection for novel inhaled products and stringent “weight of evidence” approach for generic inhalers which requires the demonstration of in vitro, pharmacokinetic, and clinical equivalence between test and reference devices to support generic product approval. In relation to the equivalence study they performed, the authors go on to state that “in this context, ignoring a two-fold difference in respirable dose between VHCs, as in the present study, is difficult to rationalise. Harmonisation of regulatory guidelines across regions would seem desirable.”

It is interesting to note that the regulatory review process for devices occurs through a different body than the pMDI drug products they are to be used with. A medical device should be developed following design controls and applicable regional and International Organization for Standardization (ISO) standards. Depending upon the device classification in a given market, approval is requested or self-certification provided. Although, for example, in Canada, there is a standard for the testing and performance of VHCs [51]; it is not known to what extent all commercially available chambers satisfy the standard as such data are not required in order to gain a registration. This may lead to substandard VHCs being available for patient use without meeting minimal quality and performance standards.

## 7. Education

Ongoing patient education is a critical factor of correct inhaler technique, adherence, and disease control. A single event of instruction with the prescription of an inhaled medication and/or spacer or VHC is not sufficient to ensure correct inhaler technique. For example, Kamps et al. [52] demonstrated that three comprehensive instruction sessions increased the number of patients demonstrating correct inhalation technique of pMDI plus spacer from 57.4% to 97.9%. Amin et al. [53] assessed patient and physician confidence in the usage of inhalers used to treat COPD. They found that approximately 30% of patients had low confidence in their ability to use their inhalers and that low confidence was associated with lower adherence and poor COPD-related health status.

Education on the correct use of VHCs with pMDIs is important to avoid the potential for new errors introduced with the VHC. These may include as incorrect device assembly, incorrect inhalation after actuation (e.g., waiting too long to inhale), and actuating more puffs than required. [4].

Each VHC device comes with directions for use [31, 38]. Generally, spacers should be positioned before actuating the pMDI, and movement of the spacer should also be avoided, as this will reduce the drug available for inhalation due to impaction on the sides of the spacer wall. Patient education must be specific to the pMDI and the VHC used [4].

Clinicians can support patients in several ways:

- Check inhaler technique often.
- Keep devices consistent when changing or adding medications, that is, try not to mix pMDI and DPI devices. Each new pairing of pMDI and VHC requires instruction specific to the devices being used.
- Use appropriate training aids for encouraging slow inhalation with pMDI devices [14].
- Ensure that VHCs are used with pMDIs by infants and children and by patients with poor coordination or inhaler technique.
- Ensure that VHCs are used when prescribing corticosteroids with pMDI to reduce oropharyngeal side effects and absorption via the gut.
- Ensure that an appropriate antistatic VHC is prescribed to prevent losses of medication to chamber walls.
- Instruct patients and caregivers to clean and use VHCs according to the PIL [31, 38].
- Ensure that VHCs with facemasks for infants and young children fit correctly with a good seal to the face, and that parents/caregivers understand the importance of no leaks [31].
- A dedicated inhalation indicator is available in one brand of VHC (AeroChamber Plus Flow-Vu). This can help provide visual feedback with respect to correct inhalation and facemask seal. *In vivo* studies show that the use of a chamber with the Flow-Vu Inhalation Indicator was associated with improved satisfaction and caregiver quality of life [54].

## 8. Summary

Inhaled aerosol therapy remains the cornerstone of effective treatment of asthma and COPD. While the medications themselves have not changed dramatically over the past decades, the delivery devices have changed. Technology has allowed the development of more efficient and user-friendly inhalers. Nevertheless, incorrect inhaler technique remains a significant barrier to many users of inhaled medications. The most common errors reported for the use of pMDIs are lack of coordination between actuation and inhalation, halting inhalation when the cool spray hits the back of the throat, not holding the breath long enough (>5 seconds) after inhalation, no exhalation prior to actuation, and not shaking the suspension prior to use. Valved holding chambers confer distinct advantages to the first two challenges. VHCs allow users to approach inhalation of aerosol medication as a two-step process: actuation into to chamber, followed by inhalation from the VHC mouthpiece. Technology has also allowed the development of more effective VHCs. There are now antistatic chambers, better valves, more effective facemasks, and other innovations that help deliver the intended dose of medication. VHCs have been proven to improve pMDI medication delivery to the lungs, reduce oropharyngeal deposition, and help users overcome challenges in coordinating pMDI actuation with inhalation [8]. Moreover, newer VHCs with multiple advances (antistatic chamber and inhalation indicators) have been reported to improve asthma control, reduce the rate of exacerbations, and improve quality of life [36, 54]. VHCs are not all the same, and also are not interchangeable. Ongoing education is critical to ensure that users are consistently able to use their inhalers.

## Figures and Tables

**Table 1:** Valved holding chambers available in Canada.

Device	Manufacturer	Material	Antistatic	Volume
A2A Spacer	Clement Clarke, UK	Plastic	Low static	210 ml
AeroChamber Plus Flow-Vu	Trudell Medical International, Canada	Plastic	√	149 ml
Aerochamber Plus Z STAT®	Trudell Medical International, Canada	Plastic	√	149 ml
InspiraChamber®	InspirRx Inc., USA	Plastic	√	Not available
Leverhaler™	BirdSong Medical	Plastic	—	Not available
Life Brand Inhalation Chamber	ProtecSom, France	Plastic	—	Not available
LiteAire®	Thayer Medical	Cardboard	—	160 ml
OptiChamber Diamond®	Philips Respironics, USA	Plastic	√	140 ml
ProChamber™	Philips Respironics, USA	Plastic	—	145 ml
RespiChamber®	Trudell Medical International, Canada	Plastic	Static resistant	149 ml
Space Chamber Plus™ compact	Medical Developments International	Plastic	√	160 ml
